# Micronutrient levels and nutritional status of school children living in Northwest Ethiopia

**DOI:** 10.1186/1475-2891-11-108

**Published:** 2012-12-13

**Authors:** Bemnet Amare, Beyene Moges, Bereket Fantahun, Ketema Tafess, Desalegn Woldeyohannes, Gizachew Yismaw, Tilahun Ayane, Tomoki Yabutani, Andargachew Mulu, Fusao Ota, Afework Kassu

**Affiliations:** 1 Department of Medical Biochemistry, University of Gondar, College of Medicine and Health Sciences, Gondar, Ethiopia; 2 Department of Microbiology, Immunology and Parasitology, University of Gondar, College of Medicine and Health Sciences, Gondar, Ethiopia; 3 Department of Pediatrics, Addis Ababa University, Addis Ababa, Ethiopia; 4 Department of Chemical Engineering, University of Tokushima, Tokushima, Japan; 5 University of Tokushima, Institute of Health Biosciences, Tokushima, Japan

**Keywords:** School children, Nutritional status, Micronutrients, Gondar, Ethiopia

## Abstract

**Background:**

Several micronutrients are essential for adequate growth of children. However, little information is available on multiple micronutrient status of school children in Ethiopia. The present study was designed to evaluate the relationship between multiple micronutrient levels and nutritional status among school children.

**Method:**

In this cross-sectional study, anthropometric data, blood and stool samples were collected from 100 children at Meseret Elementary School in Gondar town, Northwest Ethiopia. Serum concentration of magnesium, calcium, iron, copper, zinc, selenium and molybdenum were measured by inductively coupled plasma mass spectrometer. Anthropometric indices of weight-for-age, height-for-age and BMI-for-age were used to estimate the children's nutritional status. Stool samples were examined by standard microscopic methods for intestinal parasites.

**Results:**

The prevalence of stunting, underweight, wasting and intestinal parasitoses among school children was 23%, 21%, 11% and18%, respectively. The mean serum levels of magnesium, calcium, iron, copper, zinc, selenium and molybdenum were 2.42±0.32 (mg/dl), 15.31±2.14 (mg/dl), 328.19±148.91 (μg/dl), 191.30±50.17 (μg/dl), 86.40±42.40 (μg/dl), 6.32±2.59 (μg/dl), and 0.23±0.15 (μg/dl), respectively. Selenium deficiency, zinc deficiency and magnesium deficiency occurred in 62%, 47%, and 2% of the school children, respectively. Height-for-age showed significant positive correlation with the levels of copper and molybdenum (*p* = 0.01) and with the levels of magnesium (*p* = 0.05).

**Conclusion:**

Deficiencies of selenium and zinc were high among the school children although the deficiencies were not significantly related with their nutritional status. The prevalence of both malnutrition and intestinal parasitism was not negligible. These calls for the need to undertake multicentre studies in various parts of the country to substantiate the data obtained in the present study so that appropriate and beneficial strategies for micronutrient supplementation and interventions on nutritional deficiencies can be planned.

## Introduction

There is a continuing worldwide effort focused on the complete eradication of poverty and hunger [1]. However, undernutrition is still a major public health problem especially in Sub-Saharan Africa [2]. In Ethiopia, child malnutrition continues to be a major public health problem [3].

Undernourished children are more likely to develop severe infections secondary to compromised immune responses [4]. Undernutrition influences several aspects of immunity, including cell-mediated immune responses [5], cytokine production [6] and antibody responses [7] particularly those that require T cell support [8]. The high prevalence of bacterial and parasitic diseases in poor countries contributes greatly to undernutrition [9].

Children are most vulnerable to undernutrition due to low dietary intake, inaccessibility to food, inequitable distribution of food within the household, improper food storage and preparation, dietary taboos and infectious diseases [9]. Especially, micronutrient deficiencies are a result of inadequate intake or inefficient utilization of available micronutrients due to infections and parasitic infestations [4]. However, information on the serum levels of multiple micronutrients in human biological tissues is scarce. For many essential elements, baseline levels in the general population, and especially in children, are lacking [10].

The levels in children are of particular interest since adequate intake of micronutrients is of great importance for the well being, proper development, and functioning of the body starting from fetal life and throughout childhood. They have been implicated to play important roles in immuno-physiologic functions [11]. For example, zinc is an integral part of more than 200 enzymes and has significant task in nucleic acid metabolism, cell replication, tissue repair, and growth [12]. The antioxidation functions of selenium in glutathione peroxidase are essential in protecting the biological system from oxidation caused by peroxides [13]. Superoxide dismutases, which usually contain copper and/or zinc, act as antioxidants against superoxides [13]. Iron carries oxygen to the cells and is necessary for the production of energy, the synthesis of collagen, and the functioning of the immune system [14-16] and copper is required with iron for synthesis of hemoglobin. It works with many enzymes such as those involved in protein metabolism and hormone synthesis [16]. Calcium plays an important role in muscle contraction and regulation of water balance in cells. Modification of plasma calcium concentration leads to the alteration of blood pressure. Magnesium has been known as an essential co-factor for many enzyme systems. It also plays an important role in neurochemical transmission and peripheral vasodilation [17].

Micronutrient deficiencies can affect all age groups, but young children are most at risk, particularly in the developing world [18]. According to previous estimates, micronutrient deficiency accounts for approximately 7.3% of the global burden of disease [10]. Although several studies have documented the status of one or two micronutrients among children [19-26], little information is available on multiple micronutrient status of school children. The present study was, therefore, designed to evaluate the relationship between nutritional status and level of multiple micronutrients in Ethiopian school children.

## Methods

### Study area and subjects

In this cross-sectional study, 100 students were selected by simple random sampling at Meseret Elementary School in Gondar town. Gondar town is located in the Northwestern part of Ethiopia with population of 200,000 [27]. None of the school children participated in this study received any of micronutrient supplementation.

### Ethical considerations

The study was reviewed and approved by the Institutional Review Board (IRB) of the University of Gondar. Informed written consent was obtained from parents or legal guardians.

### Nutritional assessment

Body weight was determined to the nearest 0.1 kg on an electronic digital scale and height was measured to the nearest 0.1 cm. Overweight (> + 1SD BMI-for-age z score), obesity (> + 2SD BMI-for-age z score), thinness/wasting (< −2SD of BMI-for-age z score), underweight (< −2SD of weight-for-age z score) and stunting (< −2SD of height-for-age (HAZ) z score) were defined according to the WHO and USCDC references [28]. Since weight-for-age (WAZ) is inadequate indicator for monitoring child growth beyond pre-school years due to its inability to distinguish between relative height and body mass, therefore, BMI-for-age is recommended by the WHO and USCDC to assess thinness/wasting in school-aged children and adolescents [29].

### Collection of stool specimens and examination for helminths

Stool samples were collected following standard procedures in clean leak-proof stool cups. Just after collection they were examined by two senior clinical laboratory technicians independently for intestinal parasites following direct and concentration methods at Gondar University Hospital Parasitology Laboratory [30]. All children who were found to be positive for intestinal parasites were given the appropriate anti-parasite chemotherapy by a medical doctor.

### Determination of levels of trace elements in serum

Blood specimens were taken with minimal venostasis after overnight fasting for the measurement of micronutrients by phlebotomists. The venous bloods were drawn into sterile non-contaminated polypropylene tubes (Becton Dickinson, Franklin Lakes, NJ, USA); All tubes were kept in dark cool boxes (0-4°C) and transported to the Gondar University Hospital. Sera were separated from cells by centrifugation at 4000 g for 10 min at 4°C, within 4 hours. Aliquots of sera were stored at −70°C until analysis. For the determination of trace elements, sera were kept on dry ice and brought to the University of Tokushima, Japan.

Concentration of trace elements in serum was determined using an Inductively Coupled Plasma Mass Spectrometer (ICP-MS) (model 8500, Schimadzu, Tokyo, Japan), at Department of Analytical Chemistry, the University of Tokushima, Japan following previously published procedures [31,32]. In brief, serum sample (200 μl) was aliquoted in to teflon tube and covered with teflon ball. After adding 1 ml of concentrated HNO_3_ (Wako Pure Chemicals, Japan), the tube was heated on an aluminium heating block (IWAKI, Asahi Techno Glass, Japan) at 120°C for 5 hours. The sample was further heated almost to dryness at 200°C after removing the teflon ball. Finally, the residue was dissolved with 2 ml of 0.1 M HNO_3_ which contained 10 ng/ml internal standard elements (In, Re, and Tl). The diluted serum solution was used for analysis of the elements in ICP-MS. Commercially available single element standard solutions (1000 μg/ml) were purchased from Wako Pure Chemicals (Osaka, Japan) and used for standardization of calibration curves. The result was expressed in mg/dl for calcium and magnesium, and in μg/dl for copper, iron, zinc, selenium and molybdenum.

### Statistical analysis

Data were analyzed using SPSS version 13 statistical package (SPSS, Inc., Chicago, IL, USA). A one-sample Kolmogorov-Smirnov test was used to assess whether the data were normally distributed. All micronutrients values in serum were normally distributed and hence no transformation was done. Comparisons of serum values of the trace elements among students were made using one-way-ANOVA. Post hoc Tukey test was used to determine which pairs of means differ significantly. Cut off value for magnesium, calcium, iron, copper, zinc, selenium and molybdenum was defined at their serum levels of 1.8 mg/dl, 8.4 mg/dl, 60 μg/dl, 75 μg/dl, 75 μg/dl, 7 μg/dl and 0.02 μg/dl, respectively [33]. Pearson’s test was used to assess the correlation between two continuous variables. Statistical significance was assigned for *p* values less than 0.05. The z score values for height-, weight- and BMI-for-age relative to the WHO 2007 reference were calculated using Epi Info and WHO Anthro Plus softwares [34]. The z score values relative to the USCDC 2000 reference were calculated by the SPSS files provided by the USCDC [35].

## Results

The study sample consisted of 100 elementary school children between 10 to 14 years of age (mean age 12.1±2.4). Majority of the school children were males (52%). Intestinal helminthic parasites were detected in 18% of the school children (Table 1). *Ascaris lumbricoides* (10, 55.6%) was the predominant parasite identified followed by hookworm (6, 33.3%) and *Trichuris trichuria* (2, 11.1%).

**Table 1:** Demographic data of school children at Meseret Elementary School, Gondar, Ethiopia

**Features**		**Number (%)**
**Age**	5-9	11 (11)
	10-14	79 (79)
	>15	7 (10)
**Gender**	Male	52 (52)
	Female	48 (48)
**Helminths infection**	Yes	18 (18)
	No	82 (82)
**Mother education**	Educated	47 (47)
	Not educated	53 (53)

The means z-scores of HAZ, WAZ and BMI-for-age of the study participants were −1.15±1.21, -1.15±1.00 and −0.72±1.39, respectively (Table 2). The means were not significantly different between females and males in all anthropometric measures used to evaluate their nutritional status. The prevalence for the respective anthropometric measures indicated that 23%, 21% and 11% of the school children respectively were stunted, underweight and wasted (Table 2). Although not statistically significant, the prevalence rates for stunting and underweight were relatively higher among females.

**Table 2:** Nutritional status of school children at Meseret Elementary School, Gondar, Ethiopia

**Anthropometric index**	**Boys (n=52)**	**Girls (n=48)**	**Total (n=100)**	**Mean difference between sexes (95% CI)**	**P value for difference (2-tailed)**
Height-for-age	−1.13±1.11	−1.18±1.33	−1.15±1.21	0.05 (−0.43-0.54)	0.8
Z score<−2SD (stunting)	10 (19.2%)*	13 (27.1%)	23 (23%)		
Weight-for-age	−1.143±0.99	−1.15±1.03	−1.15±1.00	0.003 (−0.39-0.40)	0.9
Z score<−2SD (underweight)	8 (15.4)	13 (27.1%)	21 (21%)		
BMI-for -age	−0.69±1.08	−0.76±1.68	−0.72±1.39	0.06 (−0.49-0.61)	0.8
Z score<−2SD (thinness)	6 (11.5)	5 (10.4%)	11 (11%)		

*N(%).

Table 3 shows the concentrations of serum magnesium, calcium, iron, copper, zinc, selenium and molybdenum in school children in relation to nutritional status. The mean serum level of iron was significantly lower in severely stunted compared to moderately stunted school children (*P*<0.05). There was only a significant difference among different classification of height-for age concerning the copper-to-zinc ratio *(P*<0.05). Serum concentration of calcium was significantly higher in moderately thin (wasted) school children (*P*<0.05) compared to normal. However, serum concentration of zinc was significantly lower in mildly wasted school children (*P*<0.05) compared to normal. On the contrary, severely wasted school children had significantly higher concentration of copper, although not statistically significant. As a result, the copper-to-zinc ratio was significantly higher in mildly wasted school children (*P*<0.05) compared to normal children.

**Table 3:** Levels of serum micronutrients (Mean±SD) in relation to children's nutritional status of school children, Meseret school, Gondar, Ethiopia

		**N**	***Magnesium***	***Calcium***	***Iron***	***Copper***	***Zinc***	***Cu/Zn***	***Selenium***	***Molybdenum***
**Height-for-age**	Sever	7	2.17±0.23	14.12±2.13	223.02±18.45*	152.55±32.01	84.89±29.31	1.95±0.64	8.20±1.54	0.13±0.10
	Moderate	15	2.47±0.28	15.32±2.00	399.05±161.45	189.14±50.50	86.05±56.29	3.10±1.91	6.58±2.45	0.21±0.12
	Mild	32	2.39±0.27	15.16±1.87	297.80±132.46	186.89±47.88	95.10±39.92	2.23±0.95	6.23±2.64	0.21±0.13
	Normal	46	2.47±0.38	15.59±2.34	342.22±155.85	200.97±51.80	80.71±40.86	2.99±1.40	6.02±2.68	0.26±0.16
**Total**		**100**	2.42±0.32	15.31±2.14	328.19±148.91a	191.30±50.17	86.40±42.40	2.69±1.37a	6.32±2.60	0.22±0.15
**Weight-for-age**	Sever	4	2.32±0.49	14.46±2.23	215.97±36.75	172.31±36.17	73.86±29.76	2.70±1.43	7.56±2.41	0.14±0.10
	Moderate	17	2.46±0.25	15.70±2.033	375.58±174.65	195.40±60.79	78.106±44.49	3.24±1.90	5.29±1.95	0.267±0.15
	Mild	27	2.48±0.29	15.50±1.95	339.80±130.17	193.27±51.19	86.326±50.97	2.92±1.63	6.51±2.51	0.22±0.14
	Normal	52	2.39±0.35	15.15±2.28	315.30±150.93	190.40±47.72	90.13±37.99	2.39±0.90	6.47±2.79	0.23±0.15
**Total**		**100**	2.42±0.32	15.31±2.14	328.19±148.91	191.30±50.17	86.40±42.40	2.69±1.37	6.32±2.60	0 .23±0.15
**BMI-for-age**	Sever	7	2.47±0.42	15.36±1.61	333.334±166.37	214.97±69.68	86.96±57.37	3.31±1.81	6.06±2.66	0.26±0.19
	Moderate	4	2.80±0.12	18.31±0.87**	443.30±84.87	222.73±47.02	77.61±35.79	3.27±1.37	5.57±1.08	0.29±0.05
	Mild	23	2.44±0.37	15.40±2.07	324.75±153.87	187.91±45.81	63.74±32.48†	3.53±1.75‡	5.21±2.54	0.23±0.14
	Normal	66	2.39±0.29	15.09±2.16	321.86±148.36	188.07±49.36	94.78±41.96	2.30±0.99	6.78±2.58	0.22±0.15
**Total**		**100**	2.42±0.32	15.31± 2.14a	328.19±148.91	191.31±50.17	86.40±42.40a	2.69±1.37a	6.32±2.60	0 .23±0.15

^a^*P value* (one-way ANOVA) for the comparison between nutritional status.

*P<0.05 versus moderate HAZ.

**P<0.05 versus normal BMIZ.

†*P<0.05* versus normal BMIZ.

‡*P<0.05* versus normal BMIZ.

Table 4 shows the prevalence of multiple micronutrient deficiencies. In this study, 80% of the school children had two or more coexisting micronutrient deficiencies. Ten percents of these children had three coexisting micronutrient deficiencies. Zinc deficiency occurred in 47% of the school children, 62% had selenium deficiency and 2% had magnesium deficiency. Deficiency for both selenium and zinc was observed in 34% of the school children (data not shown).

**Table 4:** Levels of serum Micronutrients (Mean±SD) and proportions deficient children (%) in relation to children's sex, Meseret school, Gondar, Ethiopia

**Analytes**	**Boys N=52**		**Girls N=48**		**Sexes combined Deficiency (%) N=100**
	**Mean±SD (Range)**	**Deficiency (%)**	**Mean±SD (Range)**	**Deficiency (%)**	
*Magnesium*	2.43±0.38 (1.50-3.68)	2 (3.8)	2.42-0.24 (1.98-2.91)	-	2 (2)
*Calcium*	15.42±2.28 (9.35-20.52)	-	15.19±1.99 (11.77-20.11)	-	-
*Iron*	337.60±167.15 (74.49-845.51)	-	317.99±127.22 (116.34-578.04)	-	-
*Copper*	194.46±50.35 (118.24-313.02)	-	187.88±50.27 (107.94-324.96)	-	-
*Zinc*	84.37±42.45 (30.74-211.64)	27 (51.9)	88.61±42.68 (27.75-191.25)	20 (41.7)	47 (47)
*Cu/Zn ratio*	2.78±1.37 (0.90-6.56)	-	2.59±1.38 (0.93-6.86)	-	-
*Selenium*	6.07±2.47 (1.70-12.57)	33 (63.5)	6.59±2.73 (1.71-12.89)	29 (60.4)	62 (62)
*Molybdenum*	0.22±0.14 (0.03-0.59)	-	0.24±0.16 (0.05-0.63)	-	-

Bivariate correlation analysis showed a significant correlation between z-scores of height-for-age and the levels of magnesium (r=0.212, *p<0.05*), copper(r=0.275, *p<0.01*) and molybdenum(r=0.275, *p<0.01*). No significant correlation was found between the levels of micronutrients and the other anthropometric variables (Table 5).

**Table 5:** Correlation between micronutrients and nutritional status in the school children, Meseret school, Gondar, Ethiopia

**Trace elements**	**Height-for-age**	**Weight-for-age**	**BMI-for-age**
*Magnesium (mg/dl)*	0.212^*^	0.003	−0.189
*Calcium (mg/dl)*	0.161	−0.026	−0.150
*Iron (μg/dl)*	0.139	0.033	−0.035
*Copper (μg/dl)*	0.275^**^	0.033	−0.224
*Zinc (μg/dl)*	−0.020	0.092	0.086
*Cu/Zn ratio*	0.126	−0.145	−0.244
*Selenium (μg/dl)*	−0.159	0.018	0.115
*Molybdenum (μg/dl)*	0.275^**^	0.083	−0.130

*Correlation is significant at the 0.05 level (2-tailed).

**Correlation is significant at the 0.01 level (2-tailed).

## Discussion

Malnutrition, protein-energy malnutrition and micronutrient deficiencies continue to be major health burdens in developing countries, particularly in sub-Saharan Africa. It is globally one of the most common risk factor for illness and death, with hundreds of millions of pregnant women and young children particularly affected [36]. For children in developing countries, malnutrition is a considerable health problem with prevalence rates estimated to range from 4% to 46% with 1% to 10% severely malnourished [37]. The results of this study show that the prevalence of stunting observed among school children was 23%, which was in agreement with a finding from the study conducted among preschool children (24%) in northwest Ethiopia [38]. However, it was much lower compared to previous findings in Gumbrit (50%) in Ethiopia [39]. Higher prevalence of stunting were observed among school children in Tanzania (42.5%) [40], and in Malaysia (40.2%) [41].

The prevalence of stunting remains high in the area and the fact that the prevalence of stunting is much higher than that of underweight and wasting confirms that the major problem is chronic malnutrition. Since, stunting is a type of chronic malnutrition which begins in childhood, supplementing the infants and children with quality complementary food after 6 months of age and at least until age 36 months is required so as to minimize the long-term negative consequences of chronic undernutrition. In addition, investment in sustainable food-based strategies is urgently needed to combat hunger and micronutrient deficiencies [42].

In this study, the prevalence of underweight (21%) was lower than previous reports from north-western Ethiopia [38,39]. Both stunting and underweight were worsened as the study population got older, particularly for boys. This may lead to delayed onset of puberty in the boys. In addition, wasting which is usually caused by a relatively recent illness or food shortage was lower than stunting or underweight indicating that chronic malnutrition is more prevalent in Ethiopia than acute malnutrition.

In the present study, although not statistically significant, a positive correlation was observed between height-for-age z-score and serum iron levels (r=0.139, *p*>0.05). It was also demonstrated that severely stunted school children had low serum concentrations of iron when compared to normal children. This observation was also observed by other authors [43]. Less intake, poor absorption and the systemic effect of infection and utilization of iron by microorganisms for its growth and multiplication may be responsible for their lower iron status [44]. We did not observe iron deficiency in school children irrespective of sex and helminths infection. A previous study involving children in Ethiopia included a thorough assessment of dietary intake and showed that dietary iron was adequate [45]. Some crops, notably *teff*, a staple dish of many people in the study area, are high in iron [46] and fermented *enset* may increase non-heme iron absorption [47]. Moreover, intake of meat which is a source of heme iron in urban areas of the country is good [48]. Heme iron is not only well absorbed than non-heme from plant source food, but also has an enhancing effect on absorption. because of exposure to high iron intake. Non-nutritional factors may be responsible for the anaemia seen in parts of the country.

Magnesium is important in maintaining several cellular functions as it is a natural activator of most enzymes. Magnesium deficiency frequently develops in a wide variety of clinical conditions such as protein–energy malnutrition malabsorption, hypoalbuminaemia, sepsis, hypothermia, etc., conditions that are commonly seen in children in developing countries [17]. In the current study, deficiency in magnesium was observed in the school children of the present study, as 2% of them had its serum levels <1.80 mg/dl, particularly in boys. However, this prevalence is much lower than the 20.7% [19] and 51.9% [49] deficiency reported in Mexican and Vietnamese children. In addition, consistent with previous study in India [50], serum magnesium levels had significant positive correlations with height-for-age. Lower serum magnesium levels in malnourished children may be due to inadequate intake, malabsorption, diarrhoea, and infection.

The present study demonstrated that normal and high calcium levels were common in school children, deficiency occurred in none of study subjects. This is not in agreement with reports from India and Nigeria [13,14]. A possible explanation for the high serum calcium in our study area may be due to high calcium intake and sun exposure. The staple dish of many people in the study area and its environs is a pancake named *enjera* made from a cereal called *Teff* (*Eragrostisteff*) which has higher calcium than those of wheat, barley, or sorghum [51]. On the other hand, Ethiopia is located in the tropics in the horn of Africa between 3º and 15º N, 33º and 48º E where there is a large amount of sun exposure. When sunlight is plentiful, relatively high serum 25-hydroxyvitaminD3 may give rise to higher serum calcium levels [52]. Ultraviolet light is essential in this reaction. It is worth mentioning that, during infection, macrophages and other immune cells can express 1α-hydoxylase, the enzyme that converts circulating 25(OH) D3 into 1,25(OH) D3, the active form of vitamin D [53] and increased 1, 25(OH) D3 synthesis may further contribute to increased serum calcium level.

The high prevalence of zinc deficiency among the children has a far-reaching implication, as zinc is an important element performing a range of functions in the body. Zinc is a co-factor for the synthesis of a number of enzymes, DNA, and RNA [12]. Zinc deficiency has been associated with poor growth in childhood, reduced immuno-competence, and increased infectious disease related morbidity [52,54]. The findings of this study were in agreement with previous studies which have demonstrated the existence of zinc deficiencies among children of school age and early adolescence [55-57]. Several studies globally have documented the relationships between lowered zinc concentrations during childhood and morbidity from infectious diseases and the effect on cognitive development [58].

According to WHO, when the prevalence of zinc deficiency is greater than 20%, intervention to improve zinc status is recommended [59]. As a result, the study recommends planning of sustainable community-based intervention strategies to improve the zinc status of school children through zinc supplementation and fortification of staple foods with zinc are recommended. These interventions are imperative in view of the well-known adverse consequences of zinc deficiency to the health and quality of life of school-aged children, particularly in terms of academic performance.

The mean level of copper in children of this study higher than those reported for children residing in Khartoum, Sudan [23], for 10–12 years girls of urban Yemeni [60], and for healthy Japanese children [24]. The increased copper levels in serum may be due to a non specific increase in serum concentration of cooper binding protein, ceruloplasmin during acute-phase response against a variety of infections and inflammatory conditions [23].

It is interesting to note that the determination of the copper/zinc ratio has been considered helpful in reflecting the nutritional status of zinc in the human body, better than its content in serum [61]. It was also suggested that the copper/zinc ratio have diagnostic and prognostic values; if the ratio of copper/zinc exceeds 2 (Tables 2 &3), it would indicate severity of the infection [61]. In the present study, the ratio of copper/zinc was higher in serum of school children with severe nutritional status.

Like zinc and magnesium, a significantly low level of serum selenium was observed in school children compared to the study in Iran [26]. We observed selenium deficiency in a large number of school children (62%) and A negative correlation was found between serum selenium levels and height-for-age (r=−0.159). In fact, selenium deficiency has been reported as one of the major health problems in Gondar, Ethiopia [31,32], as in Asia and Africa [62,63]. Deficiencies of selenium contribute to the prevalence and severity of iodine deficiency disorders which are the most important and well-known global nutritional problems, primarily in developing countries [18]. Selenium is an integral part of the enzyme glutathione peroxidase, which forms a major cellular defense system against oxidative injury [13,18]. Selenium deficiency has been incriminated in the causation of several diseases including malignancies [13]. The diversity of nourishment sources, regional variation and different ethnic diets makes it difficult to extend these results to the whole population; however, it appears that more work is required to define acceptable requirements for selenium and zinc intakes, the prevalence of their deficiencies, and their public health significance.

Serum molybdenum level in this study was higher among school children and its level was positively correlated to height-for-age (r=0.275, *p*<0.01). However, mean serum molybdenum concentrations did not differ significantly between different nutritional statuses. In human and animal tissues, the enzymes xanthine dehydrogenase (XD)/oxidase (XO), aldehyde oxidase (AO) and sulfite oxidase (SO) require molybdopterin as cofactor and part of the enzyme molecule [64,65]. This is the first study to demonstrate the serum concentration of molybdenum among school children in Ethiopia. More research is required. Without it, the public health significance of serum molybdenum concentration in Ethiopia children and adults will remain uncertain.

It is well known that the relationship between malnutrition and infection is an intimate one, and it is often understood that this is because of impaired immune function. In the present study, we did a stool examinations and the overall prevalence of intestinal parasitic infections amongst the school children was 18%, which is low compared to different studies conducted in different parts of Ethiopia (35.5% and 83.8%) [66,67]; the difference may be due to the fact that infection rates depend on factors such as local personal hygienic and sanitary conditions, ecology and geography, among other factors. This decline can also be attributed to the conduct of mass deworming programmes targeting under five children in many parts of Ethiopia as a component of the Enhanced Outreach Strategy (EOS) started in 2004 [68]. It is therefore suggested that intervention measures have to be strengthened to further reduce intestinal helminthic infection among children and the community. This may include: improving sanitation and personal hygiene through continuous health education, multi-micronutrient supplementation, mass deworming and periodic treatment of the children.

Finally, as summarized in Table 6, results of serum levels of magnesium, calcium, iron, copper, zinc, selenium and molybdenum among children in this study and those reported from different countries has been presented. The levels of serum magnesium, calcium, iron, copper and zinc were higher in this report than reports from other countries [19-26,55,56,60]. The average selenium level in the current study participants (6.32±2.59 μg/dl) is lower to their Iranian counterparts (7.21±1.68) [26].

**Table 6:** Comparison of the mean serum levels of micronutrients in children from different countries

**Micronutrients**	**Present work Average value (*n*)**	**Reported range of sera level (*n*)**	**Country**	**Reference**
*Magnesium (mg/dl)*	2.42±0.32	2.2± 0.4 (488)	Mexico	[19]
	^a^(1.50-3.68)	1.8±0.17(243)	Vietnam	[49]
*Calcium (mg/dl)*	15.31±2.14	9.4±0.97 (5137)	India	[20]
	(9.35-20.52)	8.96±0.48 (39)	Nigeria	[21]
*Iron (μg/dl)*	328.19±148.91	60.9±30.2 (281)	Malaysia	[22]
	(74.49-845.51)			
*Copper (μg/dl)*	191.30±50.17	119.5±34.8 (28)	Sudan	[23]
	(107.94-324.96)	111.27±21.17(114)	Yemen	[60]
		109±17 (156)	Japan	[24]
*Zinc (μg/dl)*	86.40±42.40	65.4±18.9 (247)	Uganda	[25]
	(27.75-211.64)	66.4±21.5 (149)	South Africa	[55]
		122.3±55 (902)	Iran	[56]
		92±13 (156)	Japan	[24]
*Selenium (μg/dl)*	6.32±2.59	7.21±1.68 (216)	Iran	[26]
	(1.70-12.89)			
*Molybdenum(μg/dl)*	0.23±0.15	1.75 ± 0.8	USA	[65]
	(0.03-0.63)			

a -range.

A limitation of the present study is lack of detailed information on socioeconomic status, and non-availability of data on dietary intake. Such data may provide useful information to explain the situation of micronutrient status and deficiency in the population studied.

In summary, this study shows that the serum concentration of micronutrients in school children with different nutritional status was altered. The findings of the present study also reveal a high prevalence of zinc and selenium deficiencies, individually as well as concomitantly, among the school children in Gondar. Although prevalence of malnutrition was decreasing in the area [38,39], the prevalence of both malnutrition and intestinal parasitism was not negligible in this population. These calls for the need to undertake multicentre studies in various parts of the country to substantiate the data obtained in the present study so that appropriate and beneficial strategies for micronutrient supplementation can be planned.

## Abbreviations

ELISA: Enzyme linked immunosorbent assay; WHO: World Health Organization; BMI: Body mass index; SPSS: Statistical Package for Social Science; SD: Standard deviation; IQR: Inter quartile range; HAZ: Height-for-age; WAZ: Weight-for-age.

## Competing interests

The authors declare that they have no competing interests.

## Authors’ contributions

Conceived and designed the study: AK, BA, BF, KT, DW, GY, BM, TA, TY, AM, FO. Performed the laboratory analysis: AK, TY, FO. Analyzed data: AK, BA, BF, KT, DW, GY, BM, TA, TY, AM, FO. Contributed reagents/materials/analysis tools: AK, BA, BF, KT, DW, GY, BM, TA, TY, AM, FO. Wrote the paper: BA, AK, BM. All authors have read and approved of the final version of the manuscript.
